# Supplementary material for: Neanderthal-derived variants increase SOX9 enhancer activity in craniofacial progenitors that shape jaw development
Source: Development. 2025 Nov 10;152(21):dev204779. doi: 10.1242/dev.204779 (PMC12669974; doi:10.1242/dev.204779)
Supplement: Supplementary information [file develop-152-204779-s1.pdf]

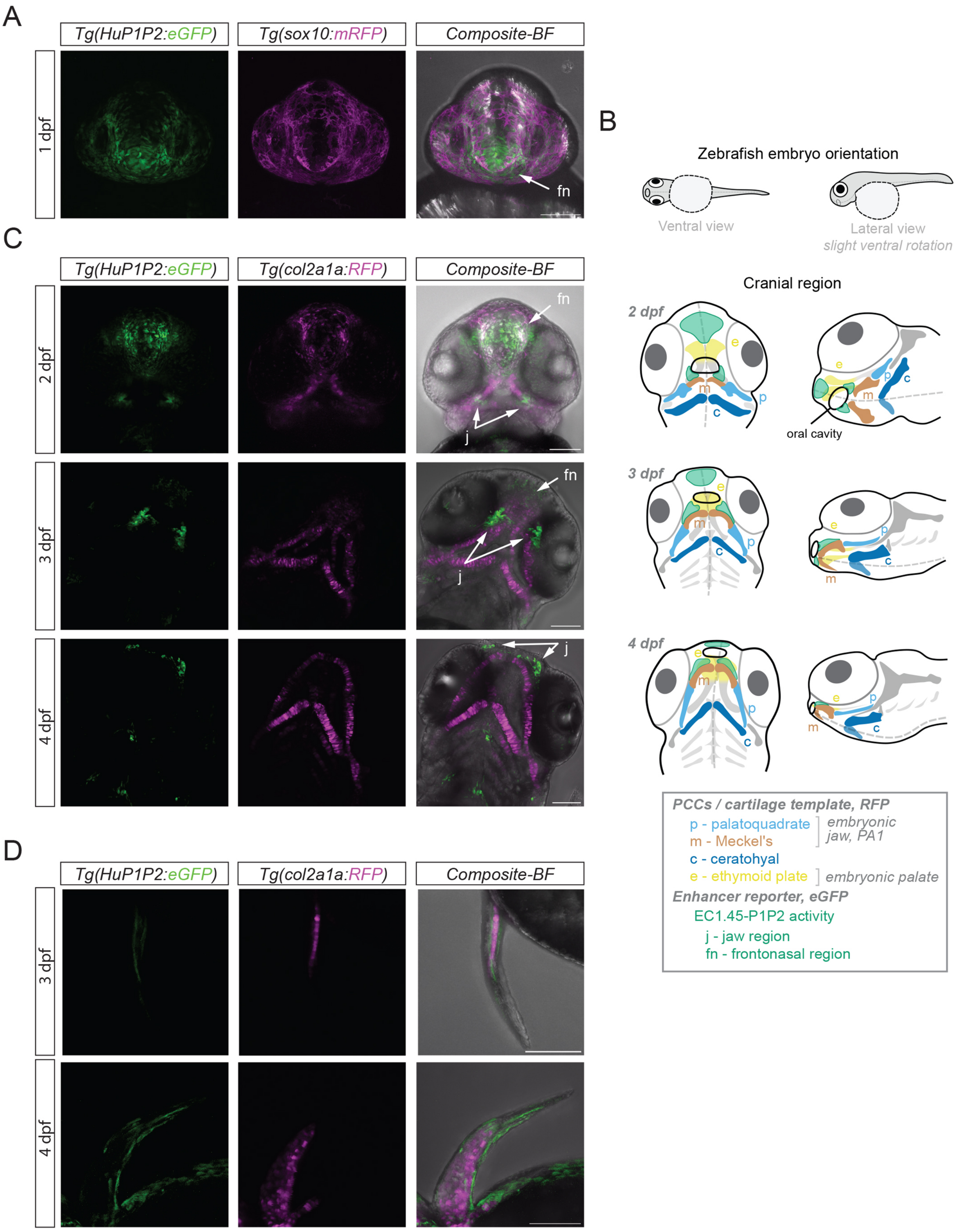
A
Tg(HuP1P2:eGFP)
Tg(sox10:mRFP)
Composite-BF
1 dpf
fn
B
Zebrafish embryo orientation
Ventral view
Lateral view
slight ventral rotation
Cranial region
2 dpf
3 dpf
4 dpf
oral cavity
C
Tg(HuP1P2:eGFP)
Tg(col2a1a:RFP)
Composite-BF
2 dpf
3 dpf
4 dpf
fn
j
D
Tg(HuP1P2:eGFP)
Tg(col2a1a:RFP)
Composite-BF
3 dpf
4 dpf
PCCs / cartilage template, RFP
p - palatoquadrate
m - Meckel's
embryonic jaw, PA1
c - ceratohyal
e - ethymoid plate
embryonic palate
Enhancer reporter, eGFP
EC1.45-P1P2 activity
j - jaw region
fn - frontonasal region

Fig. S1. EC1.45 Peak1-2 is active during embryonic facial and fin development (related to Fig. 2).

(A) Representative image of the cranial region of embryos at 1 dpf for *Tg(HuEC1.45-P1P2:eGFP)* crossed with *Tg(sox10:mRFP)*. Maximum intensity projections from confocal microscopy. Scale bar 100 µm.

(B) Schematics of zebrafish embryonic cranial region (ventral and lateral views) indicating the location of human EC1.45-P1P2 enhancer activity in relation to the *col2a1a:RFP* reporter signal in precartilaginous condensations (PCCs) which mature to form cartilage templates from 2-4 dpf. m – Meckel's; p – palatoquadrate; e – ethmoid plate; c – ceratohyal.

(C) Representative images of the cranial region of embryos at 2, 3 and 4 dpf for *Tg(HuEC1.45-P1P2:eGFP)* crossed with *Tg(col2a1a:RFP)*. Maximum intensity projections from confocal microscopy. Scale bars 100 µm.

(D) Representative images of the pectoral fin for embryos at 3 and 4 dpf for *Tg(HuEC1.45-P1P2:eGFP)* crossed with *Tg(col2a1a:RFP)*. Maximum intensity projections from confocal microscopy. Scale bars 100 µm.

Development • Supplementary information

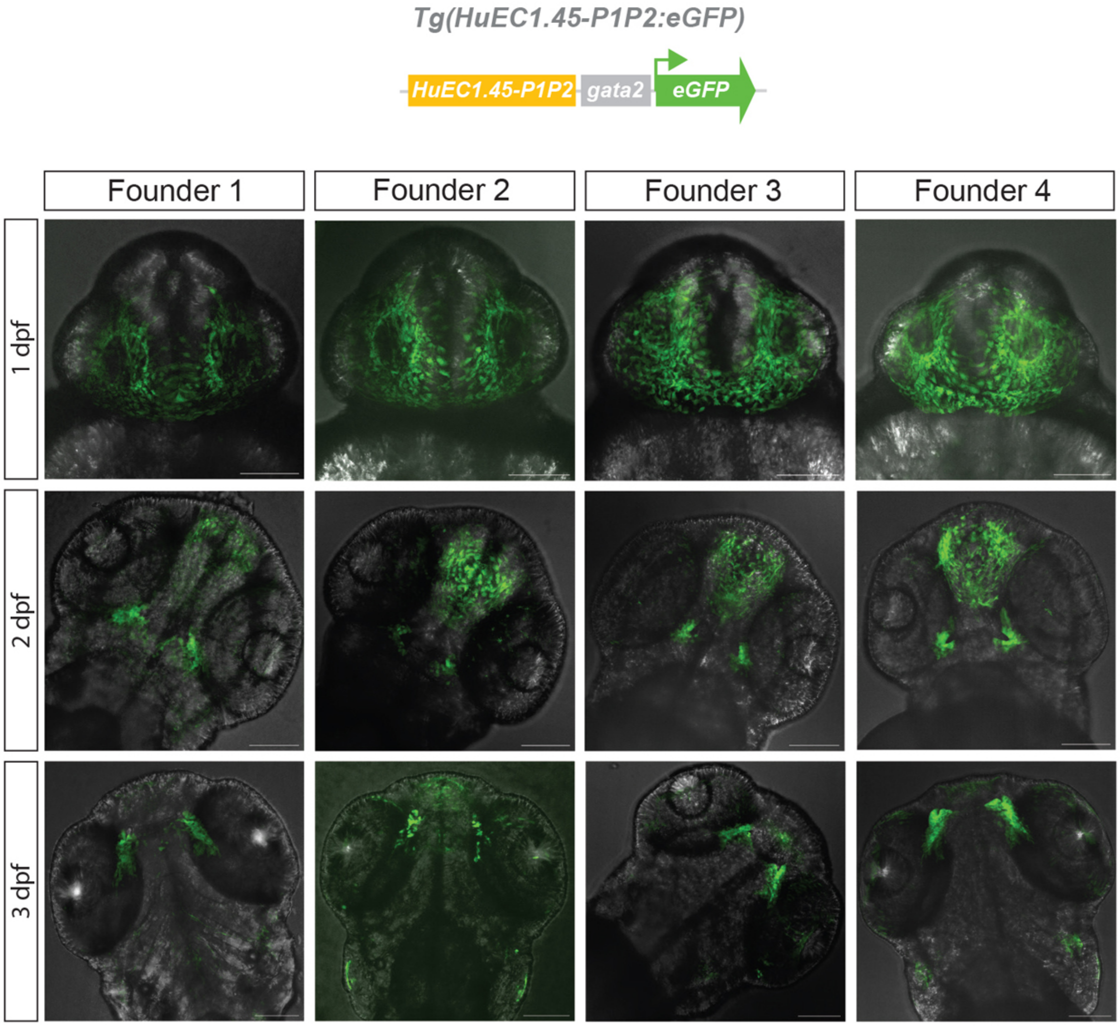

Fig. S2. EC1.45 Peak1-2 developmental enhancer activity is consistent across multiple founder lines (related to Fig. 2).

Four *Tg(HuEC1.45-P1P2:eGFP)* transgenic reporter lines derived from individual F0 founders were imaged at 1, 2 and 3 dpf demonstrating reproducibility of activity domains for EC1.45 across early zebrafish craniofacial development.

Development • Supplementary information

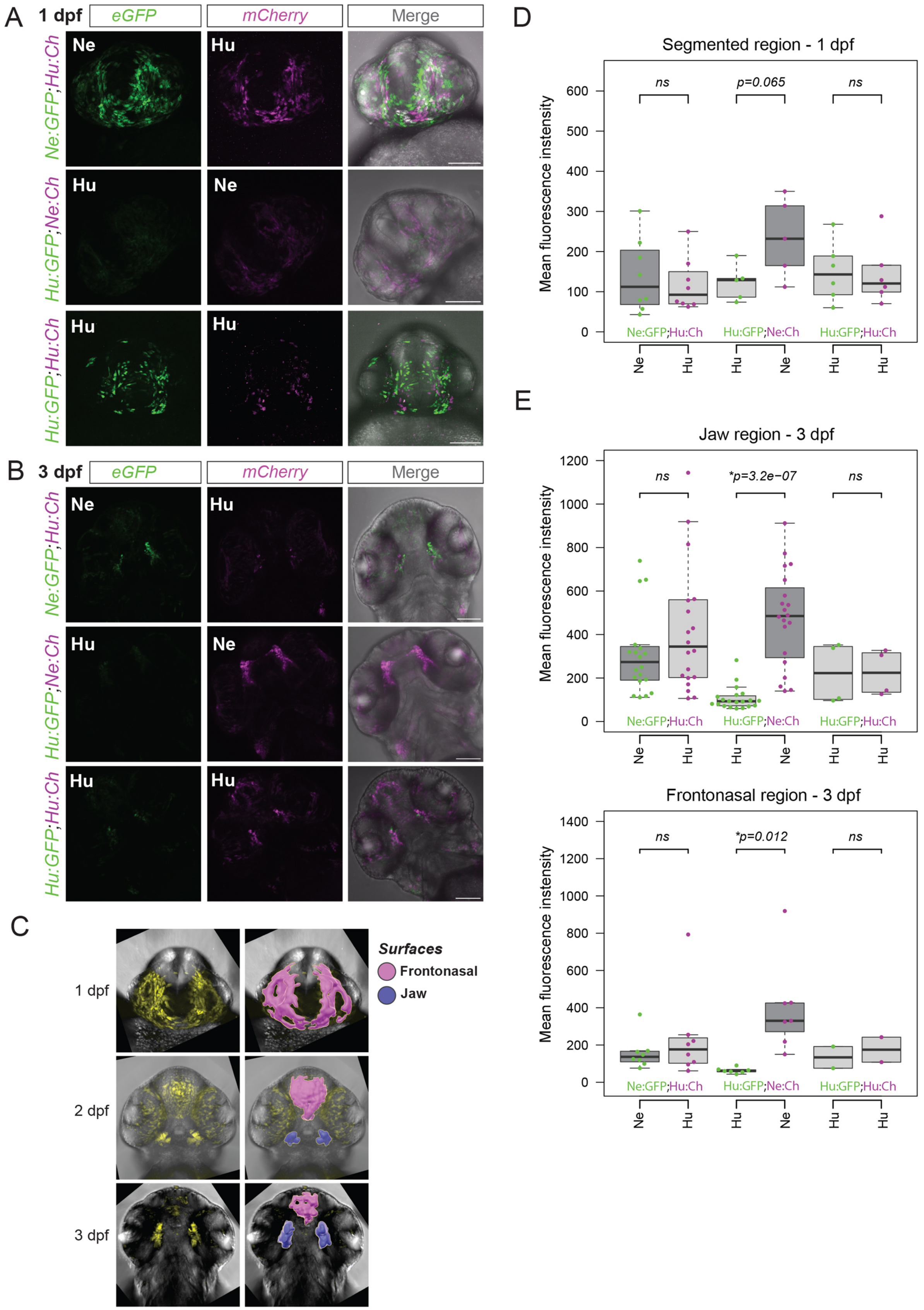
A
1 dpf
eGFP
mCherry
Merge
Ne:GFP;Hu:Ch
Ne
Hu
Hu:GFP;Ne:Ch
Hu
Ne
Hu:GFP;Hu:Ch
Hu
Hu
B
3 dpf
eGFP
mCherry
Merge
Ne:GFP;Hu:Ch
Ne
Hu
Hu:GFP;Ne:Ch
Hu
Ne
Hu:GFP;Hu:Ch
Hu
Hu
C
1 dpf
2 dpf
3 dpf
Surfaces
Frontonasal
Jaw
D
Segmented region - 1 dpf
Mean fluorescence instensity
ns
p=0.065
ns
Ne:GFP;Hu:Ch
Hu:GFP;Ne:Ch
Hu:GFP;Hu:Ch
Ne
Hu
Hu
Ne
Hu
Hu
E
Jaw region - 3 dpf
Mean fluorescence instensity
ns
*p=3.2e−07
ns
Ne:GFP;Hu:Ch
Hu:GFP;Ne:Ch
Hu:GFP;Hu:Ch
Frontonasal region - 3 dpf
Mean fluorescence instensity
ns
*p=0.012
ns
Ne:GFP;Hu:Ch
Hu:GFP;Ne:Ch
Hu:GFP;Hu:Ch

Fig. S3. Quantification of Neanderthal and human EC1.45 developmental enhancer activity at 1 and 3 dpf (related to Fig. 3).

(A) Representative confocal images (maximum intensity projections) for embryos at 1 dpf for the *Tg(Ne:GFP;Hu:Ch)*, *Tg(Hu:GFP;Ne:Ch)* and *Tg(Hu:GFP;Hu:Ch)* lines. Ventral view of cranial region. Scale bars 100 µm.

(B) Representative confocal images (maximum intensity projections) for embryos at 3 dpf for the *Tg(Ne:GFP;Hu:Ch)*, *Tg(Hu:GFP;Ne:Ch)* and *Tg(Hu:GFP;Hu:Ch)* lines. Ventral view of cranial region. Scale bars 100 µm.

(C) Illustrative images of surfaces used to quantify mean fluorescence intensity of eGFP and mCherry signal for 1, 2 and 3 dpf. At 1 dpf, the surface includes the entire domain of signal. At 2-3 dpf, the surface is separated into a frontonasal region, including some cells contributing to the developing palate (pink), and the mandible-adjacent region (blue).

(D) Quantification from (A) at 1 dpf for the entire segmented region plotted as a boxplot. P values from a Wilcoxon signed-rank test are shown.

(E) Quantification from (B) at 3 dpf for the jaw region (upper) and frontonasal region (lower) plotted as a boxplot. P values from a Wilcoxon signed-rank test are shown.

Development • Supplementary information

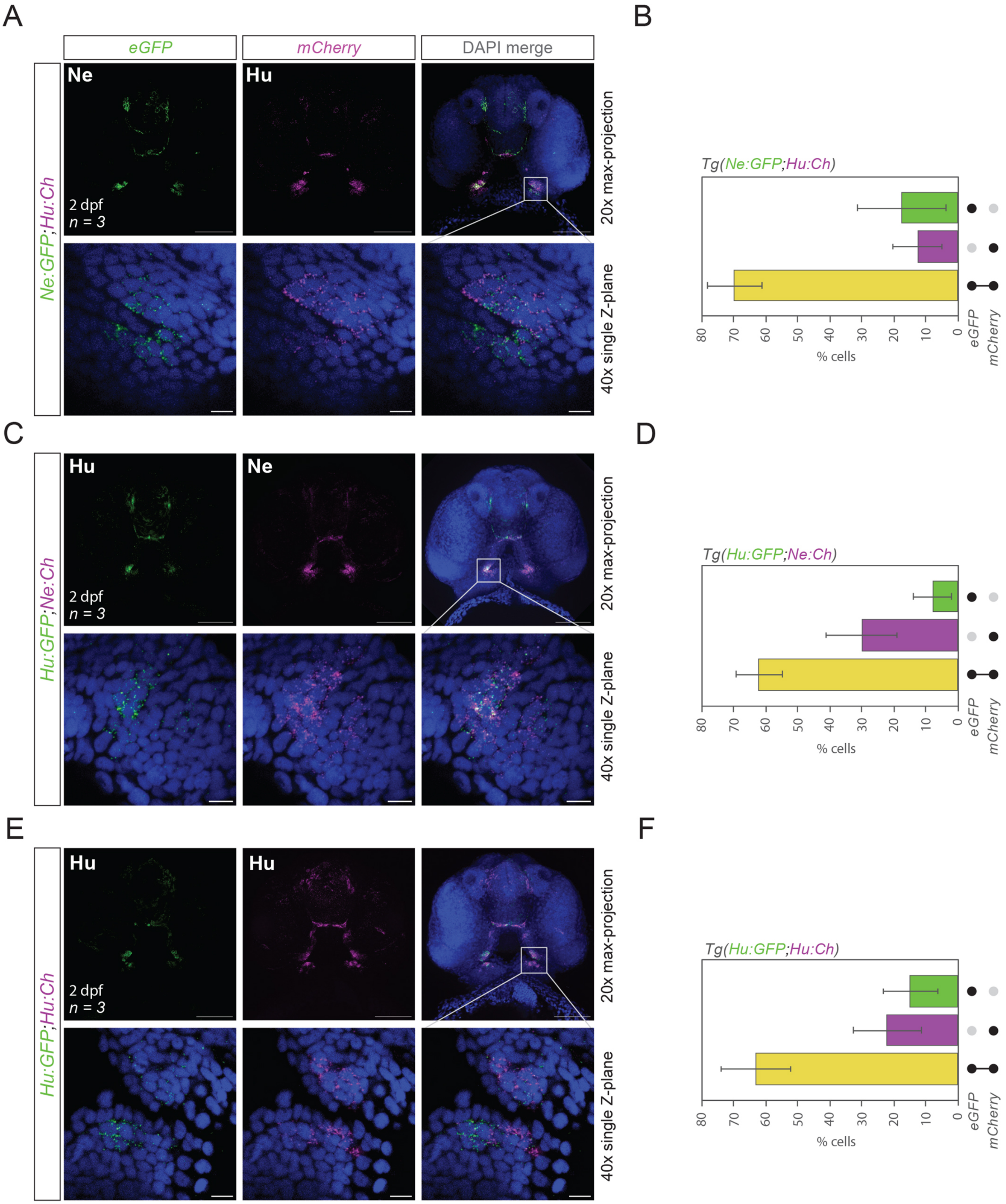

Development • Supplementary information

Fig. S4. Quantification of EC1.45 spatial activity overlap for Neanderthal and human EC1.45 using hybridisation chain reaction at 2 dpf (related to Fig. 3).

Representative HCR RNA-FISH images (A, C, E) and quantification (B, D, F) of eGFP/mCherry positive cells for *Tg(Ne:GFP;Hu:Ch)* (A-B), *Tg(Hu:GFP;Ne:Ch)* (C-D), and *Tg(Hu:GFP;Hu:Ch)* (E-F), showing co-expression of eGFP and mCherry in enhancer-active cells. Scale bars 100 μm for 20x images and 50 μm for 40x zoom. n=3 embryos from all 3 lines were used for imaging and quantification. Data plotted as bar plots with mean±s.d. error bars.

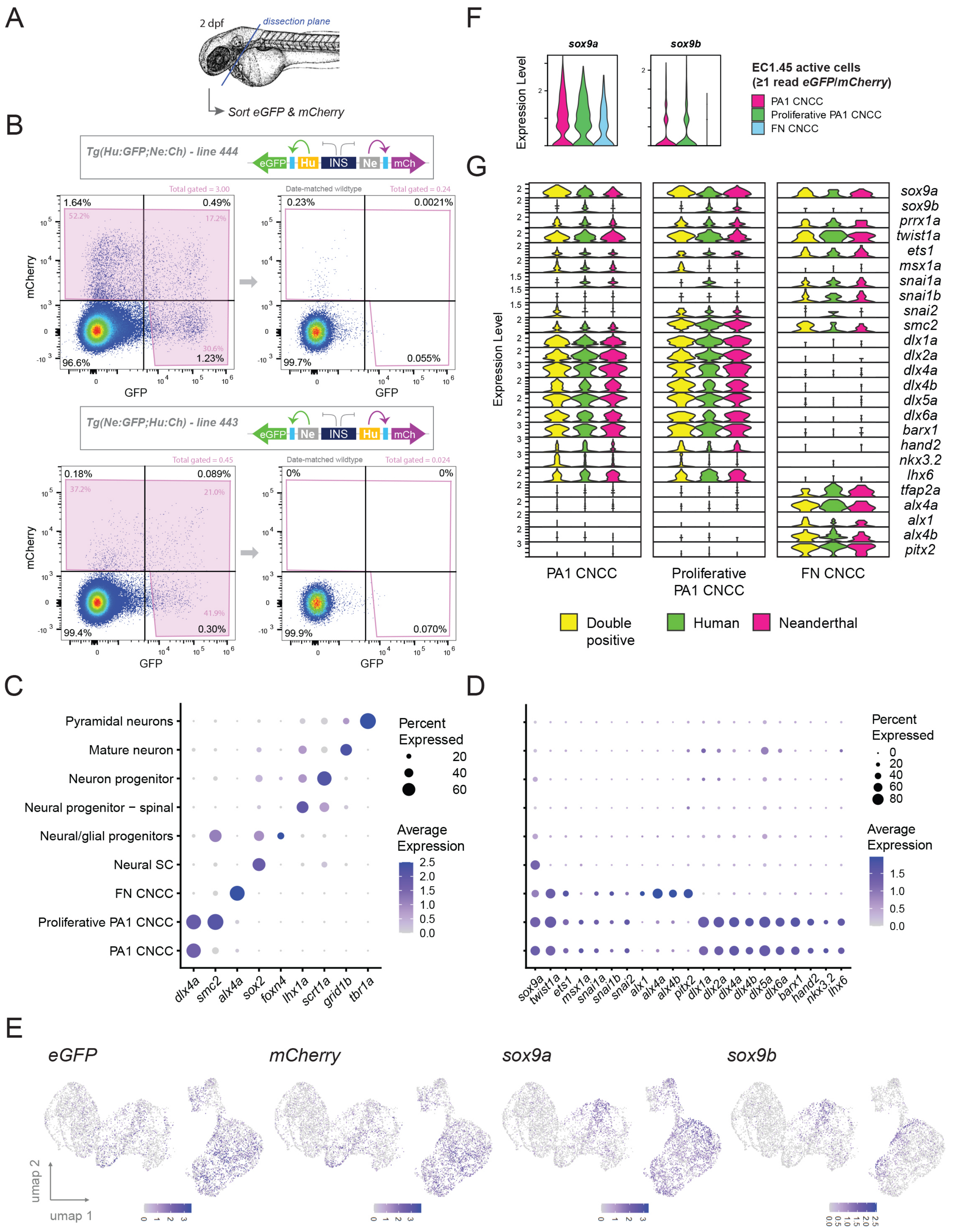

Development • Supplementary information

Fig. S5. Sorting gates for cells isolated from Q-STARZ transgenic lines, and characterisation of single cell cluster marker genes for *Tg(Hu:GFP;Ne:Ch)* (related to Fig. 4).

(A) Schematic illustrating dissection plane for isolating the cranial region from 2 dpf embryos for FACS followed by scRNA-seq.

(B) FACS plots for cells dissected from the cranial region of *Tg(Hu:GFP;Ne:Ch)* and *Tg(Ne:GFP;Hu:Ch)* transgenic lines highlighting cells that were collected for 10X scRNA-seq (pink gate, approximate gating used for sorting). Matched non-fluorescent cells from wildtype embryo cranial regions were used for setting the sorting gates.

(C) Dot plot illustrating average expression level and percentage of cells expressing markers for each cluster shown in Fig. 4A.

(D) Dot plot illustrating CNCC, frontonasal, pharyngeal arch 1 and mesenchymal condensation marker genes.

(E) Expression of *eGFP*, *mCherry*, *sox9a* and *sox9b* in single cells visualised on UMAP plots, as for Fig. 4A.

(F) Violin plots showing the expression of *sox9a* and *sox9b* in EC1.45-active cells for CNCC clusters.

(G) Violin plots showing the expression of CNCC and pharyngeal arch 1 marker genes for human (eGFP+), Neanderthal (mCherry+), or double positive cells in CNCC clusters.

Development • Supplementary information

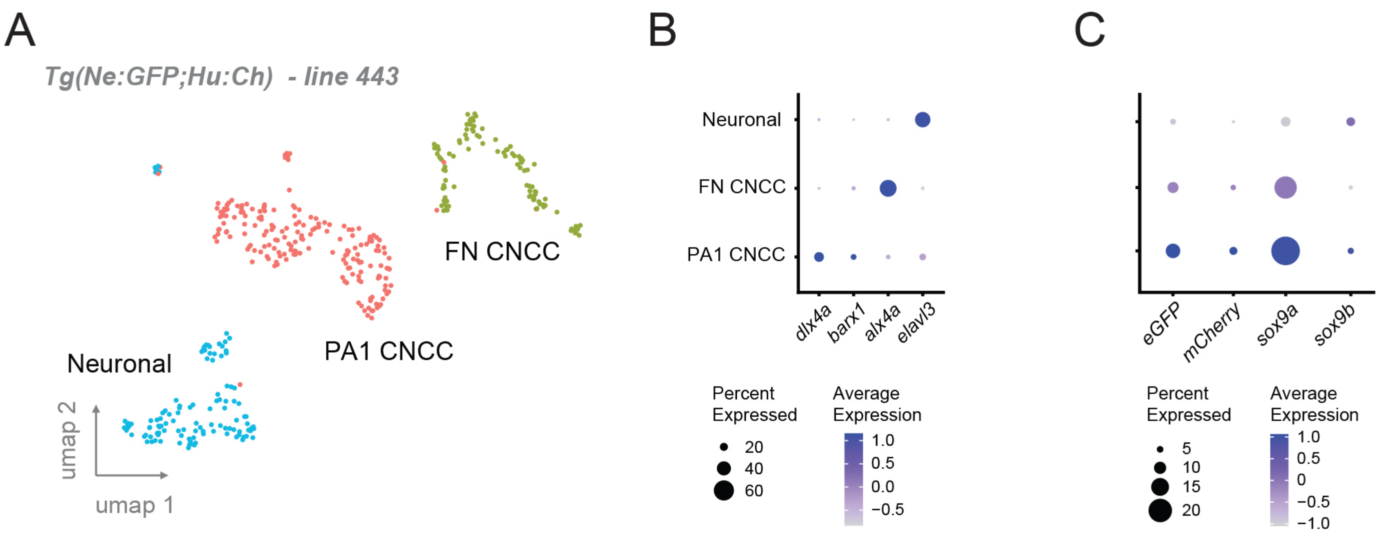

Fig. S6. Cluster identification for *Tg(Ne:GFP;Hu:Ch)* Q-STARZ transgenic line (related to Fig. 4).

(A) Cells from *Tg(Ne:GFP;Hu:Ch)* embryos at 2 dpf, visualised on a UMAP plot. Cell types were annotated to clusters aided by marker gene expression and the Daniocell atlas.

(B) Dot plot showing average expression level and percentage of *Tg(Ne:GFP;Hu:Ch)* cells expressing markers for each cluster shown in (A).

(C) Dot plot showing average expression level and percentage of *Tg(Ne:GFP;Hu:Ch)* cells expressing eGFP, mCherry, *sox9a* or *sox9b* (clusters from A).

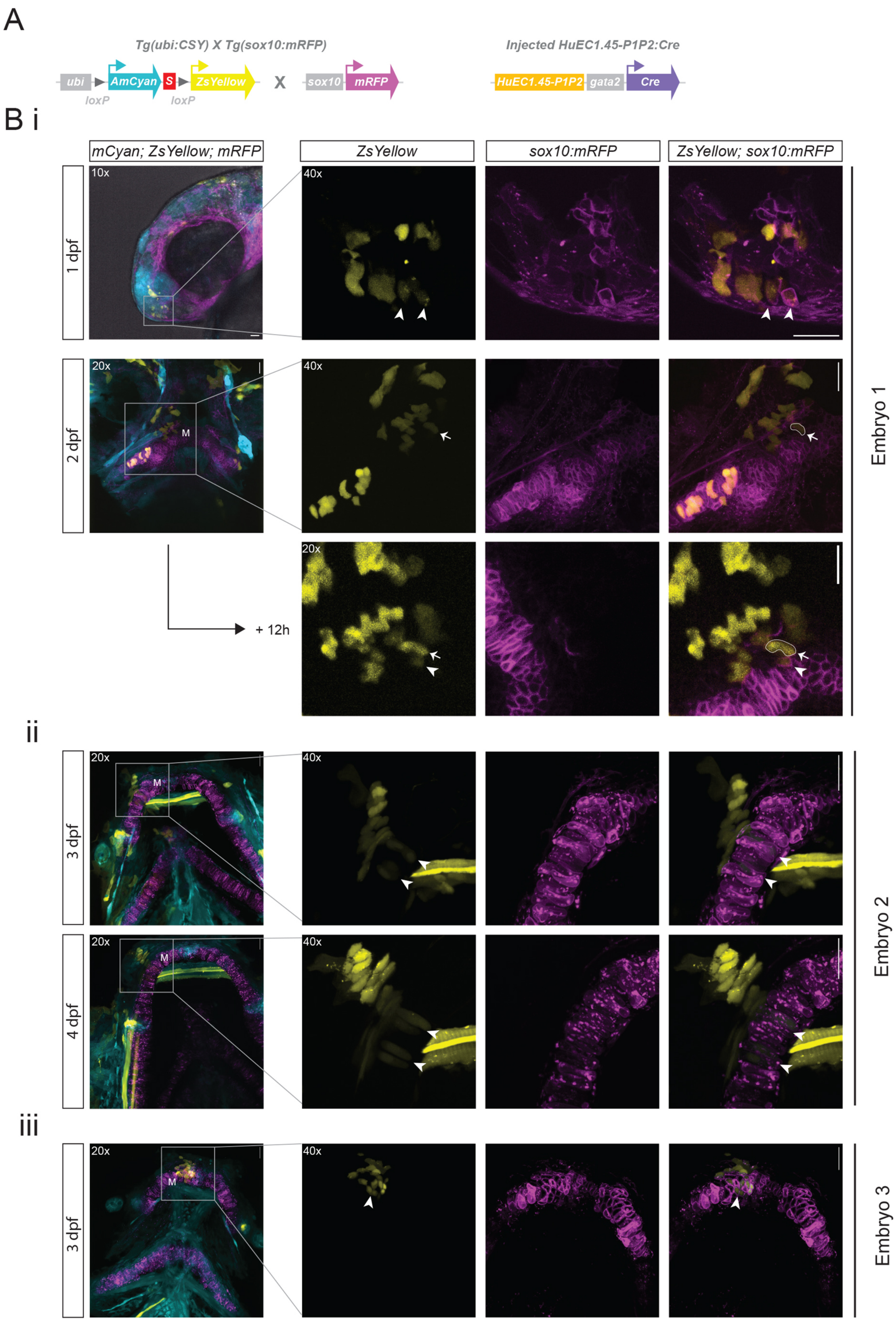

Development • Supplementary information

Fig. S7. EC1.45-active cells appear to contribute to Meckel's cartilage using Cre-mediated lineage tracing reporter.

(A) Schematic of a cross between the *Tg(ubi:CSY)* reporter line (full name *Tg(ubi:LoxP-AmCyan-LoxP-ZsYellow)*) and *Tg(sox10:mRFP)*. Resultant embryos were injected with the *HuEC1.45-P1P2:Cre* construct along with Tol2 mRNA.

(B) (i) Confocal images (maximum intensity projections) for representative embryo 1, for which cells in the frontonasal craniofacial region at 1 dpf exhibited a Cre-mediated AmCyan to ZsYellow switch (arrowheads, lateral images). At 2 dpf, ZsYellow signal is observed adjacent to the Meckel's precartilaginous condensation (PCC) (arrow). Across 12 hours of time-lapse imaging from 2 dpf, a ZsYellow-positive, sox10-positive cell is observed to appear overlapping the developing Meckel's PCC region, a white arrow highlights an outlined cell for reference which is proximal to the emerging ZsYellow-positive cell, marked by an arrowhead (see also Movie 8). M – Meckel's PCC.

(ii) Confocal images (maximum intensity projections) for representative embryo 2, for which ZsYellow-positive cells were identified at 3 dpf adjacent to the Meckel's cartilage, proximal to ZsYellow-positive, sox10-positive Meckel's chondrocytes (white arrowheads). At 4 dpf, elongation of the ZsYellow-positive chondrocytes can be observed (white arrowheads).

(iii) Confocal images (maximum intensity projections) for representative embryo 3, for which ZsYellow-positive, sox10-positive cells were identified at 3 dpf adjacent to and within the Meckel's cartilage (white arrowhead). Scale bars 20 μm. Objective used for imaging is indicated in upper left corner of images. M – Meckel's.

Development • Supplementary information

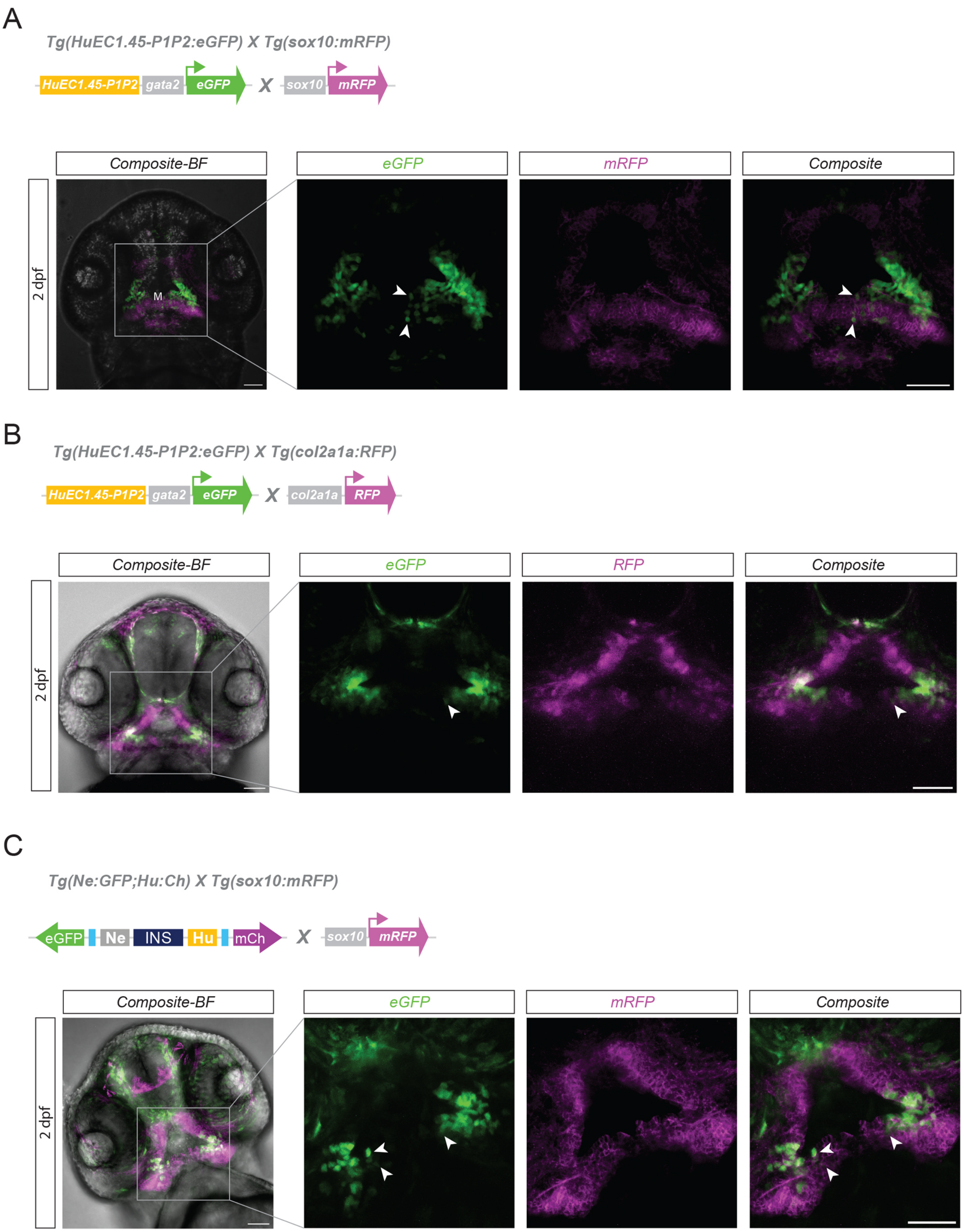

Development • Supplementary information

Fig. S8. Stable *eGFP* expression from EC1.45 enhancer reporter lines supports contribute of EC1.45-active cells to Meckel's cartilage.

(A) Representative confocal microscopy image (maximum intensity projection) showing a ventral view of the embryonic cranial region for a 2 dpf embryo from a cross between the *Tg(HuEC1.45-P1P2:eGFP)* and *Tg(sox10:mRFP)* lines. eGFP-positive, sox10-positive cells in Meckel's PCC are indicated by white arrowheads (double-positive cells within Meckel's PCC were identified for 4/4 embryos imaged).

(B) As for (A), showing a ventral view of a 2 dpf embryo from a cross between the *Tg(HuEC1.45-P1P2:eGFP)* and *Tg(col2a1a:RFP)* lines. eGFP-positive, sox10-positive cells in Meckel's PCC are indicated by white arrowheads.

(C) Representative confocal microscopy image (maximum intensity projections) showing a ventral view of the embryonic cranial region for a 2 dpf embryo from a cross between the *Tg(Ne:GFP;Hu:Ch)* and *Tg(sox10:mRFP)* lines. eGFP-positive, sox10-positive cells in Meckel's PCC are indicated by white arrowheads. Scale bars 50 µm.

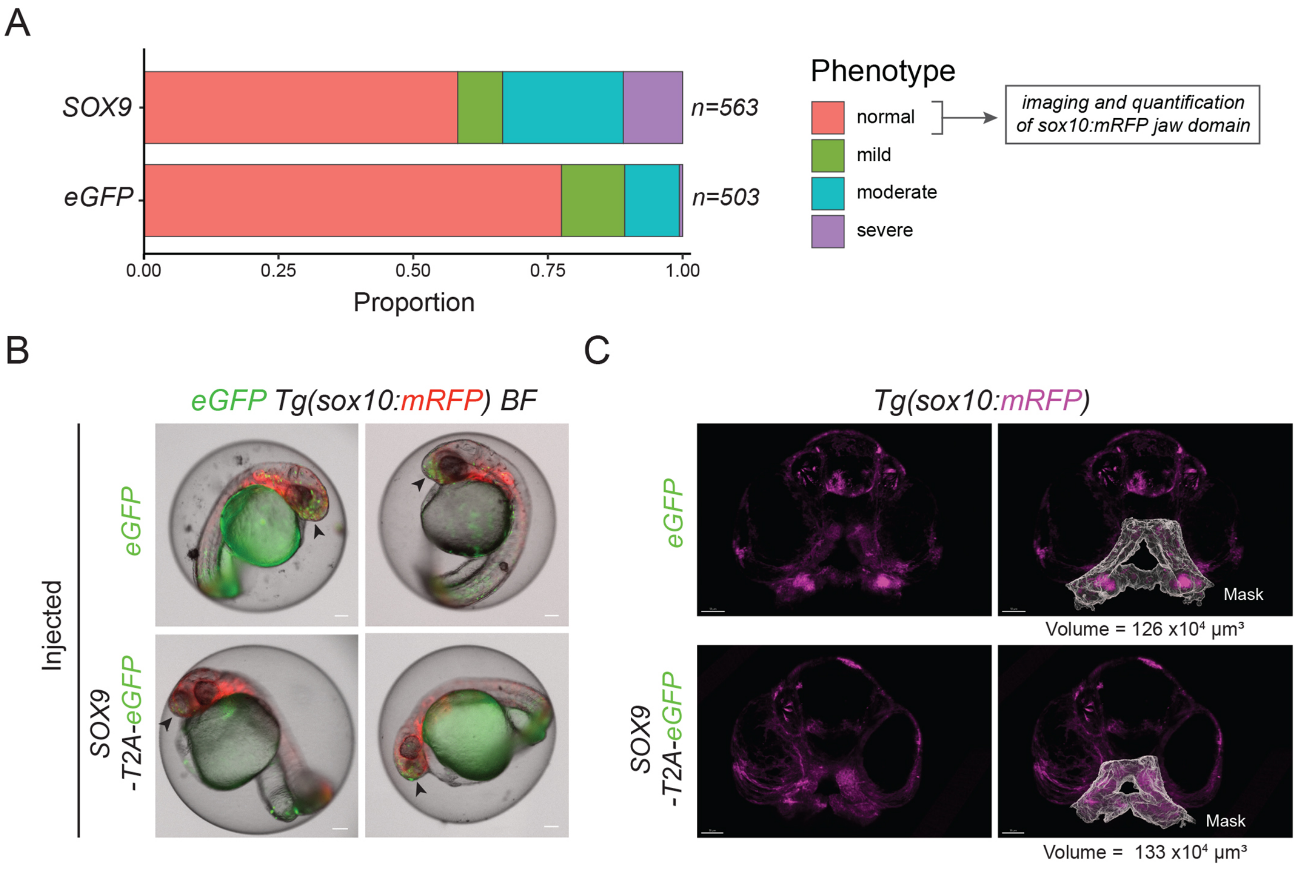

Fig. S9. Selection of normal-looking embryos from SOX9 or eGFP overexpression, representative eGFP expression in the craniofacial region and volume quantification for mesenchymal precartilaginous condensations (related to Fig. 5).

(A) Quantification of phenotypes observed at 1 dpf after injection at the 1-cell stage with either HuEC1.45-P1P2:SOX9-T2A-eGFP or HuEC1.45-P1P2:eGFP. Mild phenotypes include common abnormalities such as delayed growth or mild heart oedema, moderate phenotype indicates spinal deformities, while severe denotes embryos without observable cranial development.

(B) Images of *Tg(sox10:mRFP)* embryos injected with either HuEC1.45-P1P2:eGFP or HuEC1.45-P1P2:SOX9-T2A-eGFP at 1 dpf. eGFP expression can be seen in the developing craniofacial region (black arrowheads).

(C) Representative images of segmented regions of *sox10:mRFP* expressing cells in the developing jaw and oral-adjacent regions for either HuEC1.45-P1P2:eGFP or HuEC1.45-P1P2:SOX9-T2A-eGFP injected embryos*.* The *Tg(sox10:mRFP)* reporter was used to create a surface encompassing the mesenchymal cells of Meckel's and palatoquadrate precartilaginous condensations and the regions extending along the oral ectoderm. Scale bars 50 µm.

Development • Supplementary information

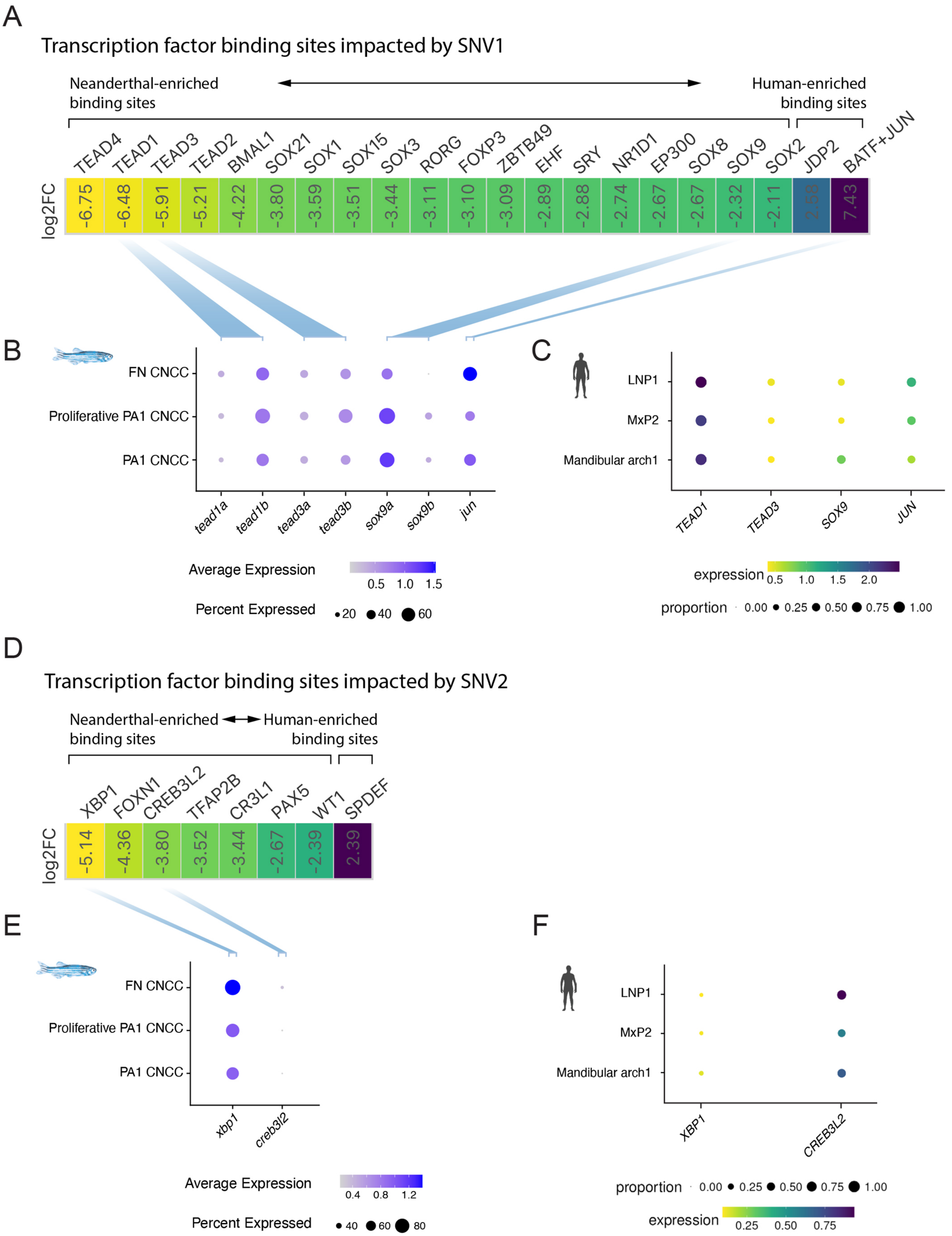

Development • Supplementary information

Fig. S10. Several candidate TFBSs are impacted by Neanderthal-derived SNVs, a subset for which the predicted TF is expressed during facial development.

(A) Transcription factor binding sites predicted to be impacted by Neanderthal-derived SNV1, ranked by log2 fold-change (log2FC) of P value for Neanderthal versus human binding.

(B) Dot plot depicting average expression for transcription factors expressed in at least 10% of cells from zebrafish scRNA-seq for three CNCC clusters (from Fig. 4A).

(C) Dot plot depicting average expression of orthologous transcription factors from (B) from human embryonic scRNA-seq craniofacial clusters (Khouri-Farah et al. 2025).

(D-F) As for (A-C) for Neanderthal-derived SNV2.

Table S1. scRNA-seq cluster marker gene expression for dual enhancer reporter.
Expression of cluster marker genes for scRNA-seq data from *Tg(Hu:GFP;Ne:Ch)* transgenic reporter embryo cranial regions at 2 dpf, called using Seurat FindAllMarkers.

Available for download at
https://journals.biologists.com/dev/article-lookup/doi/10.1242/dev.204779#supplementary-data

Table S2. A large proportion of cells from CNCC clusters exhibit fluorescent reporter expression.
Expression of eGFP (≥3 reads, green), mCherry (≥3 reads, magenta) or both (≥3 reads in total for eGFP and mCherry, yellow) across scRNA-seq clusters.

Available for download at
https://journals.biologists.com/dev/article-lookup/doi/10.1242/dev.204779#supplementary-data

Table S3. Several TFBSs gain predicted binding affinity due to Neanderthal-derived SNV1.
TFBS identified using Perfectos Ape tool for binding sites impacted by EC1.45 SNV1 (Vorontsov et al. 2015).

Available for download at
https://journals.biologists.com/dev/article-lookup/doi/10.1242/dev.204779#supplementary-data

Table S4. Several TFBSs gain predicted binding affinity due to Neanderthal-derived SNV2.
TFBS identified using Perfectos Ape tool for binding sites impacted by EC1.45 SNV2 (Vorontsov et al. 2015).

Available for download at
https://journals.biologists.com/dev/article-lookup/doi/10.1242/dev.204779#supplementary-data

Table S5. Genomic coordinates for human EC1.45 and Neanderthal variants.

Bed table outlining genomic coordinates (hg19) for human EC1.45, Peak1-2 (marked by p300 binding) and min1-2 (defined previously by deletion screening in Long et al. 2020). Genomic coordinates for Neanderthal SNVs are also shown for the hg19 genome reference.

Available for download at
https://journals.biologists.com/dev/article-lookup/doi/10.1242/dev.204779#supplementary-data

Table S6. Sequences of HCR RNA-FISH probes.

Table outlining target gene (*eGFP*, *mCherry* and *sox9a*), probe number and probe-binding sequence for HCR (Molecular Instruments, Inc).

Available for download at
https://journals.biologists.com/dev/article-lookup/doi/10.1242/dev.204779#supplementary-data

Table S7. Initial cell counts for scRNA-seq and following analysis and filtering steps.

Cell counts from scRNA-seq datasets for *Tg(Ne:GFP;Hu:Ch)* and *Tg(Hu:GFP;Ne:Ch)* dissected and sorted transgenic reporter embryo cranial regions at 2 dpf after initial cell calling and following quality control steps and filtering.

Available for download at
https://journals.biologists.com/dev/article-lookup/doi/10.1242/dev.204779#supplementary-data

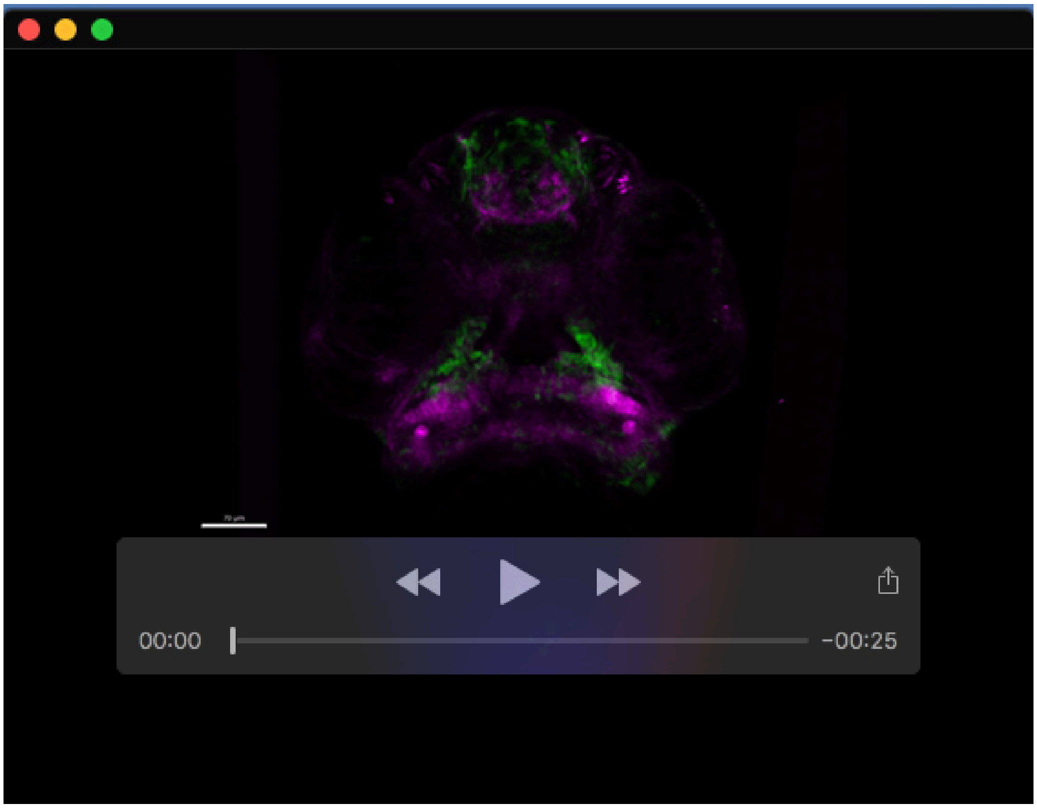

Movie 1. Cranial EC1.45 activity at 2 dpf, focusing on jaw-adjacent signal.
Animated movie of a confocal z-stack in 3D illustrating the location of eGFP-positive cells (EC1.45-activity) in relation to mRFP (developing jaw precartilaginous condensations) at 2 dpf for a cross between the transgenic lines *Tg(HuP1P2:GFP)* and *Tg(sox10:mRFP)*. Prepared in Imaris, scale bar is indicated.

Development • Supplementary information

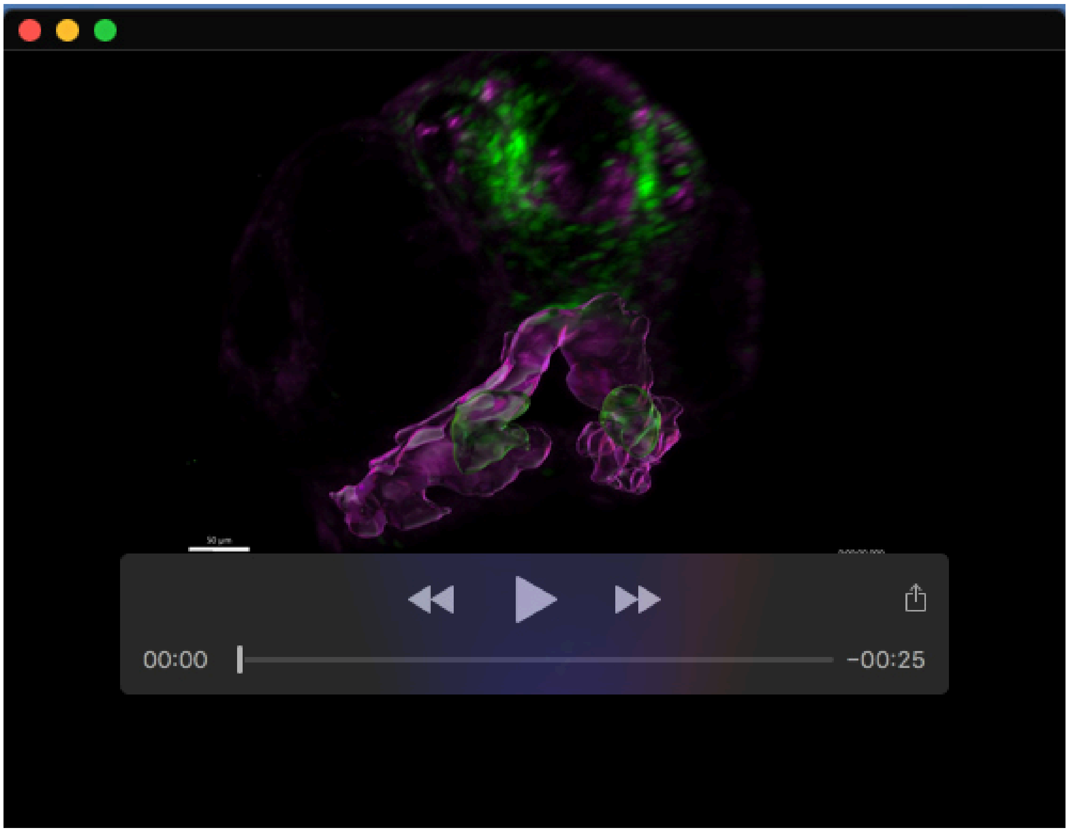

Movie 2. Surface renders of jaw-adjacent EC1.45 activity alongside *sox10* reporter marking the embryonic jaw and oral ectoderm-adjacent mesenchymal condensations at 2 dpf. Animated movie of a confocal z-stack in 3D showing eGFP-positive and mRFP-positive cells in the developing jaw region as surfaces at 2 dpf for a cross between the transgenic lines *Tg(HuP1P2:GFP)* and *Tg(sox10:mRFP)*. eGFP-positive regions form paired structures at the hinge region adjacent to the oral cavity, and proximal to the forming Meckel's precartilaginous condensation. The sox10:mRFP surface encompasses the mesenchymal cells of Meckel's and palatoquadrate precartilaginous condensations and mesenchymal cells extending along either side of the oral ectoderm. Prepared in Imaris, scale bar is indicated.

Development • Supplementary information

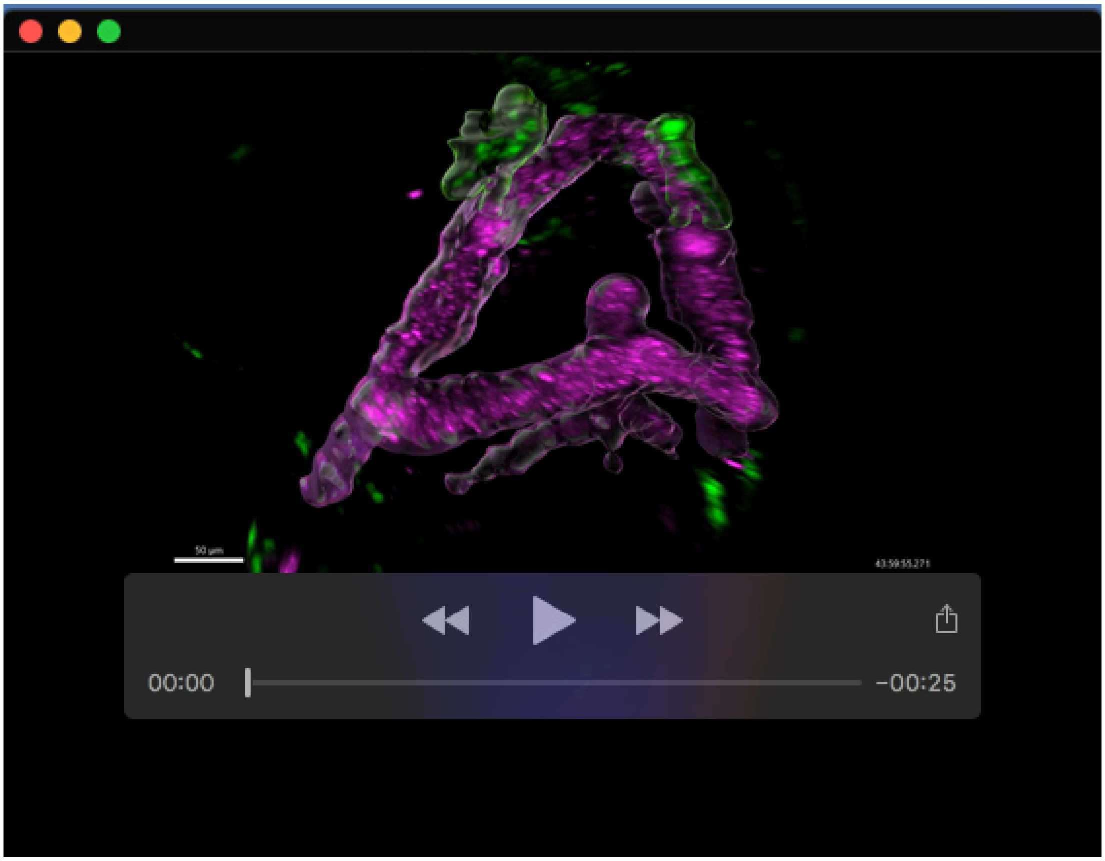

Movie 3. Surface renders of jaw-adjacent EC1.45 activity alongside *sox10* reporter marking the larval jaw and nearby cartilaginous structures at 4 dpf.

Animated movie of a confocal z-stack in 3D showing eGFP-positive and mRFP-positive cells in the developing jaw region as surfaces at 4 dpf for a cross between the transgenic lines *Tg(HuP1P2:GFP)* and *Tg(sox10:mRFP)*. eGFP-positive cells form paired regions adjacent and lateral to the Meckel's cartilage, with signal extending into and around the jaw joint between Meckel's and the palatoquadrate cartilage template (marked by sox10:mRFP). Prepared in Imaris, scale bar is indicated.

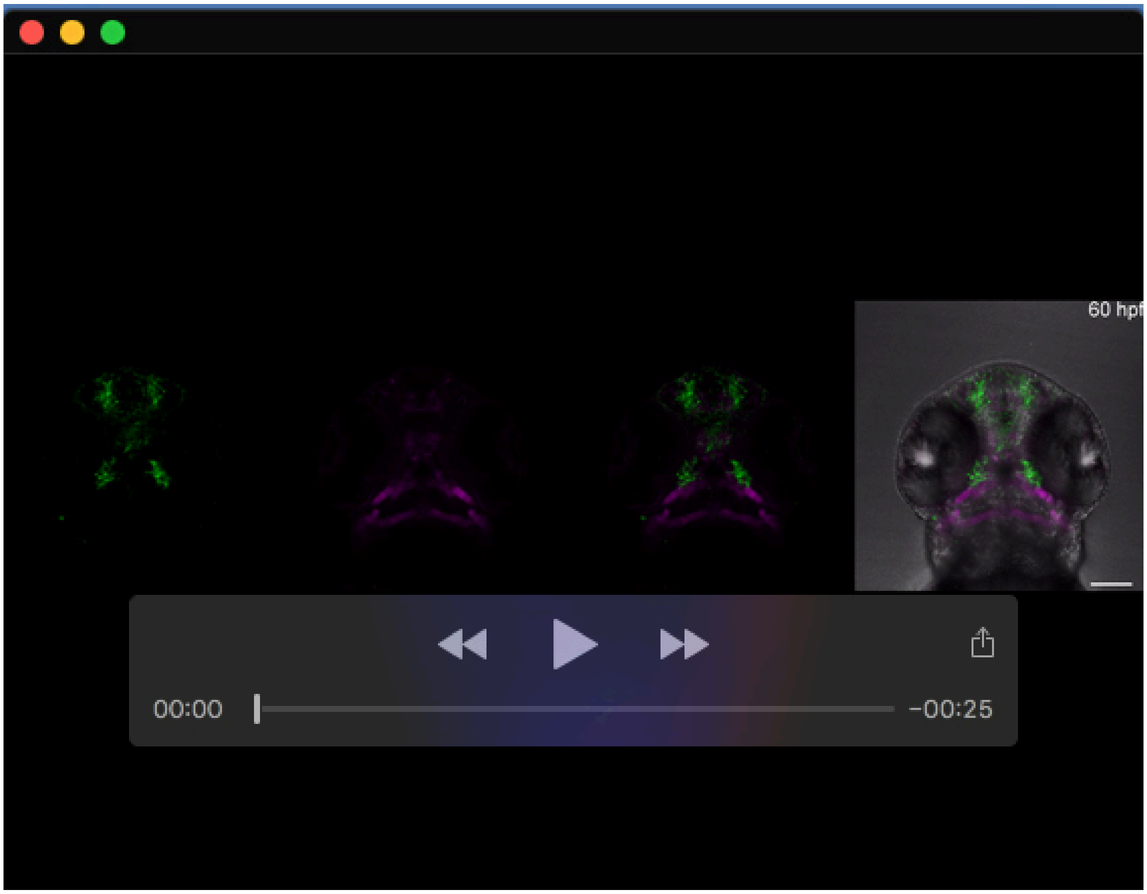

Movie 4. EC1.45 activity remains adjacent to developing Meckel's cartilage across early development.

Time-lapse movie of confocal images (maximum intensity projections) from around 2-4 dpf for an embryo from a cross between transgenic lines T*g(HuP1P2:GFP)* and *Tg(sox10:mRFP)*. Scale bar 100 μm.

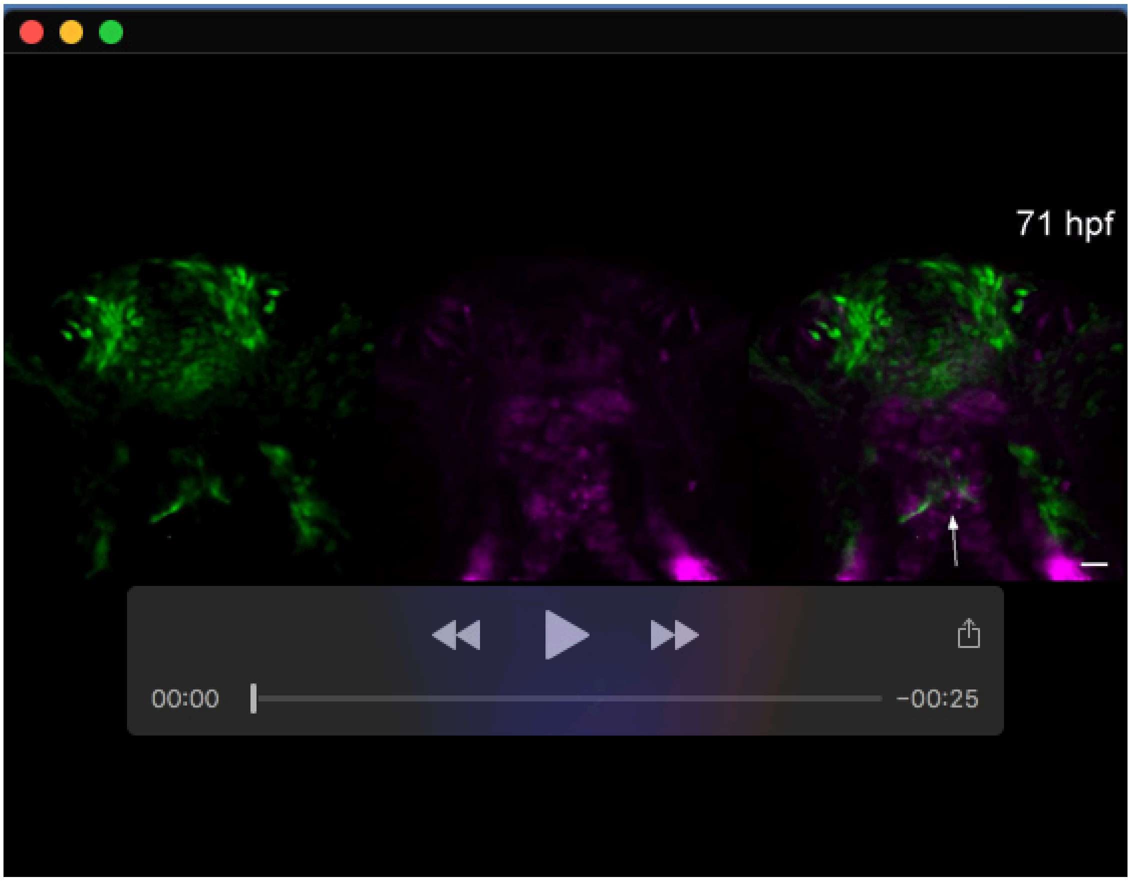

Movie 5. EC1.45-active cells are observed in the forming embryonic palate between 2-3 dpf.

Time-lapse movie of confocal images (maximum intensity projections) from Movie 4, cropped and focused on the developing embryonic palate region. White arrow indicates eGFP-positive cells in the ethmoid plate (embryonic palate). Scale bar 20 µm.

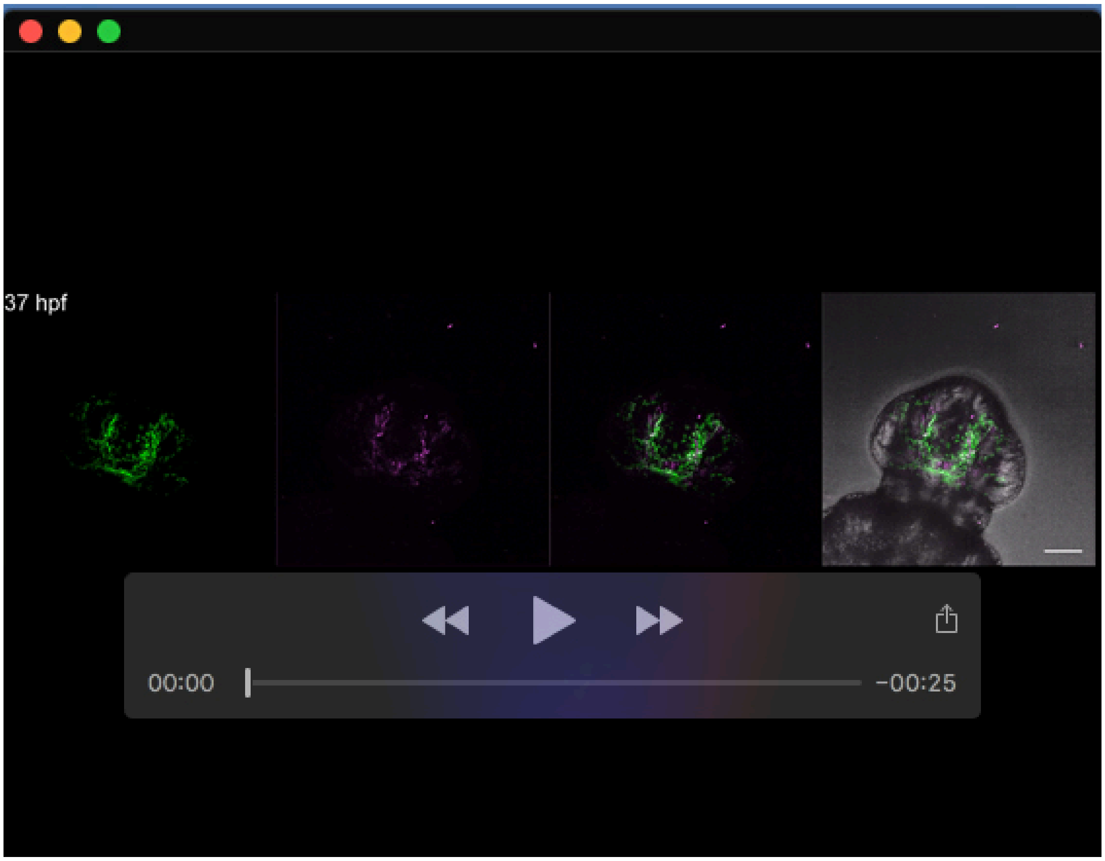

Movie 6. Human and Neanderthal EC1.45 activity is observed from 39-40 hpf in the jaw-adjacent region by live imaging.

Live imaging for *Tg(Ne:GFP;Hu:Ch)* embryo from 1-2 dpf (32-50 hpf). Arrows indicate detection of enhancer activity in the jaw region for Neanderthal EC1.45 (eGFP) and human EC1.45 (mCherry). Min/max values adjusted to clearly visualise both fluorophores over time. Scale bars 100 μm.

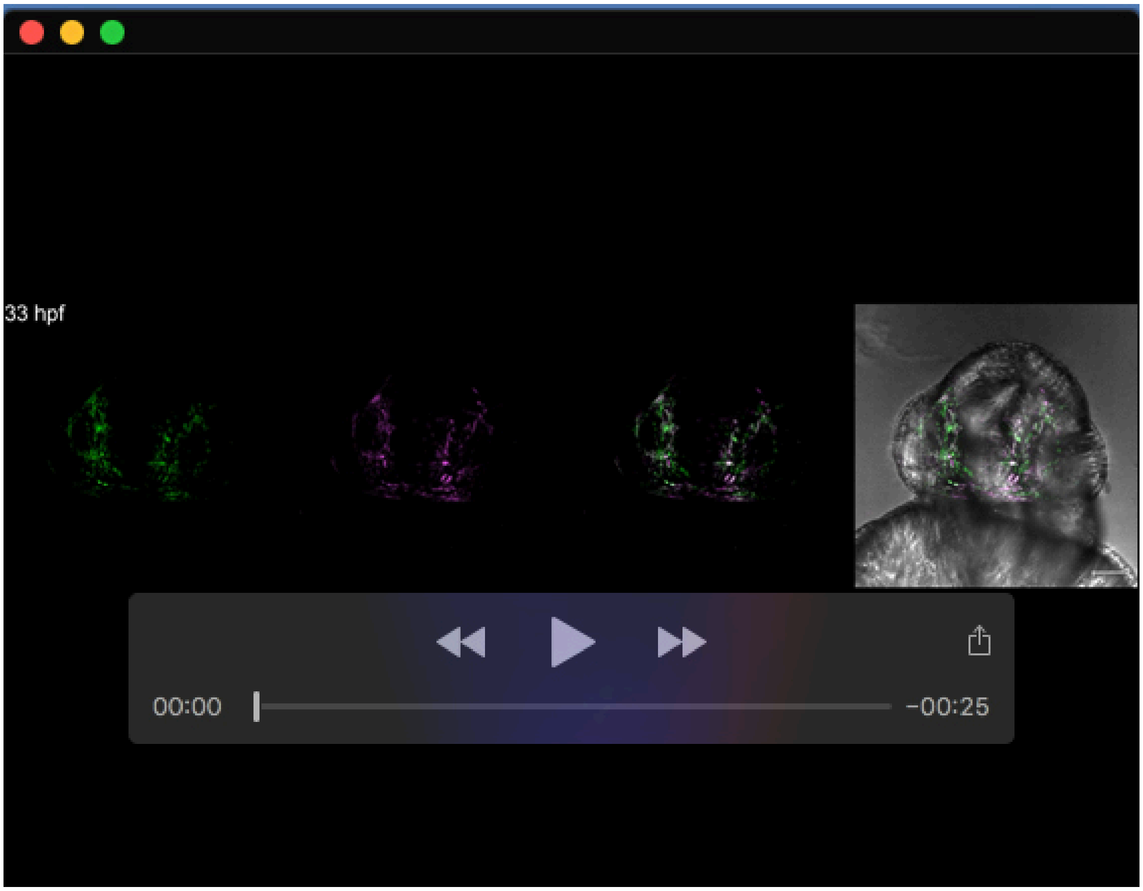

Movie 7. Human and Neanderthal EC1.45 activity is observed from 38-40 hpf in the jaw-adjacent region by live imaging.

Live imaging for *Tg(Hu:GFP;Ne:Ch)* embryo from 1-2 dpf (28-48 hpf). Arrows indicate detection of enhancer activity in the jaw region for Neanderthal EC1.45 (mCherry) and human EC1.45 (eGFP). Min/max values adjusted to clearly visualise both fluorophores over time. Scale bars 100 µm.

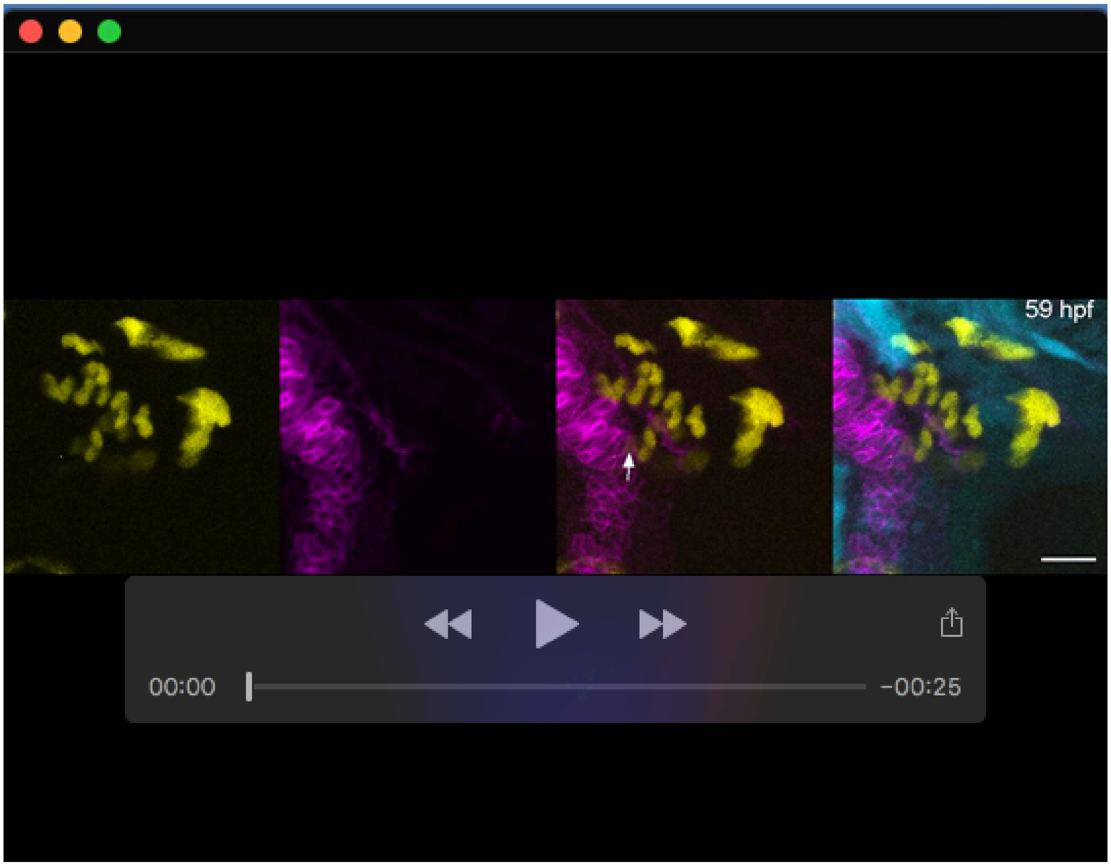

Movie 8. EC1.45-active cells appear to contribute to Meckel's cartilage formation by Cre-mediated lineage tracing.

Time-lapse movie of confocal images (maximum intensity projections) over 12 hours from 2 dpf of representative embryo 1 (see Fig. S7Bi), which exhibited an AmCyan to ZsYellow switch in the frontonasal craniofacial region at 1 dpf. ZsYellow signal was observed adjacent to Meckel's cartilage at 2 dpf, with emergence of a ZsYellow-positive, sox10-positive cell within Meckel's cartilage observed at around 60 hpf (white arrow). Scale bar 20 µm.
